# Illness Representations and Coping Strategies in Patients Treated with Deep Brain Stimulation for Parkinson’s Disease

**DOI:** 10.3390/jcm9041186

**Published:** 2020-04-21

**Authors:** Marc Baertschi, Nicolas Favez, João Flores Alves Dos Santos, Michalina Radomska, François Herrmann, Pierre R. Burkhard, Alessandra Canuto, Kerstin Weber, Paolo Ghisletta

**Affiliations:** 1 Faculty of Psychology and Educational Sciences, University of Geneva, Boulevard du Pont-d’Arve 40, 1205 Geneva, Switzerland; nicolas.favez@unige.ch (N.F.); Michalina.Radomska@etu.unige.ch (M.R.); Paolo.Ghisletta@unige.ch (P.G.); 2 Nant Foundation, Service of General Psychiatry and Psychotherapy, Avenue des Alpes 66, 1820 Montreux, Switzerland; 3 Service of Liaison Psychiatry and Crisis Intervention, Geneva University Hospitals, Rue Gabrielle-Perret-Gentil 4, 1205 Geneva, Switzerland; Joao.FloresAlvesDosSantos@hcuge.ch; 4 Division of Geriatrics, Geneva University Hospitals, Chemin du Pont-Bochet 3, 1226 Thônex, Switzerland; Francois.Herrmann@hcuge.ch; 5 Service of Neurology, Geneva University Hospitals, Rue Gabrielle-Perret-Gentil 4, 1205 Geneva, Switzerland; Pierre.Burkhard@hcuge.ch; 6 Faculty of Medicine, University of Geneva, Rue Michel Servet 1, 1206 Geneva, Switzerland; Alessandra.Canuto@unige.ch (A.C.); kerstin.weber@hcuge.ch (K.W.); 7 Swiss Distance Learning University, Überlandstrasse 12, 3900 Brig, Switzerland; 8 Swiss National Centre of Competence in Research LIVES—Overcoming Vulnerability: Life Course Perspectives, Universities of Lausanne and of Geneva, CH-1015 Lausanne, Switzerland

**Keywords:** deep brain stimulation, Parkinson’s disease, illness representations, illness perceptions, coping strategies

## Abstract

There is a debate on possible alterations of self-identity following deep brain stimulation for neurological disorders including Parkinson’s disease. Among the psychological variables likely to undergo changes throughout such a medical procedure, illness representations and coping strategies have not been the target of much research to this day. In order to remedy this, we investigated the dynamics of illness representations and coping strategies in an 18-month longitudinal study involving 45 patients undergoing deep brain stimulation for idiopathic Parkinson’s disease. Two research hypotheses were formulated and investigated through repeated measures of ANOVAs and structural equation modelling with full information maximum likelihood and Bayesian estimations. Representations of Parkinson’s disease as a cyclical condition and perception of control over the disease diminished after surgery. Use of instrumental coping strategies was not modified after deep brain stimulation. These changes were identified by SEM but not ANOVAs; their magnitude was nevertheless relatively small, implying general stability in representations. These findings suggest that psychological variables do not undergo major changes after deep brain stimulation for Parkinson’s disease.

## 1. Introduction

A chronic and degenerative condition without a widely available curative treatment, Parkinson’s disease (PD) is characterized by a complex constellation of motor and non-motor symptoms [1,2]. Preferential treatment targets motor symptoms and consists in substitutive dopaminergic therapy. Yet, when disabling symptoms persist despite optimal medical treatment, patients may be proposed to undergo deep brain stimulation (DBS), a stereotactic neurosurgical procedure during which electrodes are implanted into strategic deep nuclei of the brain [3]. DBS results in rapid motor improvement, in some cases immediately observable after the stimulation is switched on [4]. In addition, DBS allows neurologists to drastically decrease patients’ dopaminergic medication, leading to a positive impact on drug-induced motor and non-motor symptoms [5,6]. Beneficial effects on motor symptoms and medication decrease for patients treated with DBS over those only treated with medication had large size effects with a Cohen’s *d* approaching 1.35 in both cases [7].

As illustrated by the therapeutic objectives of DBS, there is a medical focus on motor symptoms in PD that underscores the under-appreciation of health professionals and researchers regarding non-motor symptoms [8]. This factual situation is nevertheless paradoxical as, in the long run, patients consider non-motor symptoms as more disabling than motor symptoms [9]. In this regard, it should be mentioned that recent research points out the importance to consider non-motor symptoms along with motor symptoms in the development of future treatments such as closed-loop DBS [10]. Among the numerous non-motor symptoms of PD identified in the literature, some are related to mental health such as depression, apathy, anxiety, or hallucinations [1,8]. Although a leading treatment like DBS provides moderate improvement (*d* ≈ 0.30) on variables related to mental health and quality of life [7], it is unsurprising that a range of non-motor symptoms remain in the patients’ clinical picture even after a medically successful DBS.

The paradox of patients experiencing a medically successful DBS, objectively assessed in measuring the pre/post-operative difference of motor functioning, but nevertheless, a psychosocially unsuccessful DBS characterized by dissatisfaction regarding the everyday life was pictured as a ‘burden of health’ by Burkhard et al. [11]. Progressively, the concept of social or psychosocial adjustment has emerged as a pivotal theme in the post-DBS rehabilitation of PD patients [12,13]. More recently, a study has provided empirical data supporting the beneficial effects of designing specific rehabilitation programs based on psychosocial adjustment [14]. In this context, researchers have suggested that the burden of normality, a model of psychosocial adjustment initially developed for epilepsy surgery [15,16], could relevantly describe the ins and outs of psychosocial adjustment difficulties throughout the DBS process provided that characteristics inherent to PD (e.g., symptoms continuing to develop post-surgery) are taken into account [17,18,19]. Among the various difficulties listed in the burden of normality regarding the post-surgical adjustment process, patients may encounter psychological changes and notably feelings of self-transformation [16]. In PD, patients have indeed reported identity-related complains following DBS surgery, such as feelings of strangeness, loss of body control and dehumanization [12,20,21,22,23], which were associated with life adjustment difficulties. As patients undergoing DBS for PD expect improvement of their motor symptoms, unexpected side effects of brain surgery leading to psychological changes would constitute, in addition to adjustment problems, an ethical issue: Indeed, such involuntary changes would possibly induce more harm than benefits and therefore be experienced negatively because they are undesired [24,25,26]. In a recent literature review, Gilbert et al. [27] have nevertheless pointed out the lack of empirical data supporting changes after DBS in variables that could labelled ‘psychological’ such as personality, self-identity, agency, authenticity and autonomy. This publication has given rise to a scientific debate that is still ongoing [28,29]. Among similar psychological types of variables, neither illness representations nor coping strategies have been widely studied in the context of DBS, notably for PD.

Both illness representations and coping strategies are psychological constructs pivotal to understand the dynamics that are in play in the patients’ subjective perception of their clinical condition. The common sense model [30,31] indeed posits that internal or external stimuli (e.g., appearance of clinical symptoms) generate cognitive and emotional representations of what is associated with a potential danger (e.g., an illness). These representations are addressed with coping strategies, whose efficiency in the symptom/illness management is appraised, leading to possible changes to better adapt the situation. Thus, patients’ subjective representations of their illness are closely intertwined with the way they attempt to cope with the stress related to their clinical condition, as both illness representations and coping strategies are likely to be modified depending on the perceived outcome on health [30,31,32]. From this perspective, one may wonder whether DBS could constitute a life experience disruptive enough to elicit psychological changes in patients; such kinds of changes would besides have a potential impact on psychosocial adjustment as illness representations and coping strategies have been associated to a variety of adaptive and maladaptive outcomes in terms of well-being and social functioning [33].

To our knowledge, no research has been published on the illness representations of patients undergoing DBS for PD. Nevertheless, based on the common sense model, one might expect that DBS would bring in changes in patients’ feelings of personal control over their symptoms. Indeed, in advanced PD, patients look to gain a certain degree of control over their symptoms, including motor and non-motor fluctuations, by managing themselves medication intake [12,22,34,35,36,37]. Yet, DBS requires active and regular neurologist intervention to adjust stimulation parameters to PD continuous development, which might lead patients to experience feelings of increased dependence on external intervention in their postsurgical disease management. In line with this, one may wonder whether DBS would lead patients to consider PD as more cyclical with regard to the regular search for stimulation adjustment. On the other hand, it is likely that PD would remain identified as such and associated with severe consequences despite the motor improvement brought in by DBS, as the diseased continues to develop and is frequently associated with psychosocial symptoms [20,23,38].

In contrast, a limited number of studies have longitudinally addressed coping strategies before and after surgery, yielding contradictory findings. While some found that patients use more frequently instrumental coping strategies (i.e., task-oriented responses, such as looking for information or for efficient treatment) before than after surgery [14,39,40], others noted that coping strategies were not employed differently over this period [41]. When observed, changes in coping strategies were associated with patients’ situation regarding their illness. Those about to undergo surgery or who could possibly be treated by DBS in the future sought out further information on this procedure, hence a more frequent recourse to instrumental strategies. Yet, patients already operated appeared to be concerned by new life issues and, accordingly, adjusted their ways of coping; they nevertheless kept using more instrumental strategies than those not selected for DBS, who employed predominantly emotional coping strategies (e.g., avoidance, emotional preoccupation) [39,40].

In light of the above, the present study aimed to address, at least partially, the current lack of empirical data regarding potential changes in the psychological experience of patients undergoing DBS highlighted by Gilbert et al. [27]. This study focused on two specific aspects, namely illness representations and coping strategies, in patients treated with DBS for idiopathic PD through an 18-month longitudinal investigation. Taking limitations of our methodological design into account, we decided to preferentially test hypotheses underlying potential pre/post-DBS changes. Thus, based on the existing literature, we assumed that patients should consider PD after surgery as more cyclical and less controllable in comparison with the pre-DBS period (Hypothesis 1). Second, as all patients included in the study knew that they were about to undergo surgery, they should have resorted to instrumental strategies more frequently before DBS than after (Hypothesis 2).

## 2. Materials and Methods

### 2.1. Procedure

Forty-five patients diagnosed with PD and treated at the Geneva University Hospitals were included between 31 January 2013 and 8 June 2017 in a global study investigating the possible determinants of quality of life after DBS. The study procedure consisted in filling out a series of self-reported questionnaires 2 weeks before DBS surgery (T0), as well as 6 (T1), 12 (T2) and 18 (T3) months after. Inclusion criteria were a DBS medical indication for idiopathic PD collaboratively established by senior neurologist, neurosurgeon, neuropsychologist and consultation-liaison psychiatrist; a sufficient French level to read and understand a series of self-administered questionnaires; and agreement to sign a consent form. Participation was proposed to all patients clinically accepted for DBS, which includes capacity of discernment, and all participants agreed with the study procedure.

The present study received approval from the Canton of Geneva ethics committee under the registration number 14-182.

### 2.2. Participants

At study inclusion, patients were aged 60.6 ± 8.6 years and most of them (60.0%) were males. The majority (66.7%) had been in a couple relationship for a long time (on average 33.1 ± 13.0 years), and 71.4% had children (1.5 ± 1.2 on average). Patients had a school background of 12.1 ± 5.3 years and had been diagnosed with PD for 9.8 ± 4.0 years. Patients score at the third part of the Movement Disorder Society Unified Parkinson’s Disease Rating Scale (MDS-UPDRS), a clinician-administered measurement of motor functioning standard in PD, was 21.105 ± 11.347 at T0, 11.419 ± 7.244 at T2 (corresponding to a 45.9% improvement), and 14.720 ± 6.921 at T3 (corresponding to a 30.3% improvement compared to T0). No measure was available at T1, post-DBS scores correspond to an on-medication and on-stimulation condition.

### 2.3. Instruments

The revised version of the Illness Perception Questionnaire (IPQ-R) [42] assesses attributes of illness representations based on the common sense model of Leventhal et al. [30,31]. This instrument proposes a self-evaluation of the main attributes defined by the common sense model, that is the identity of the illness (i.e., the observed symptoms, which may form a label—typically, the name of a disease), the possible causes of the illness, the timeline defining an acute, chronic or cyclic perception of the illness course, the consequences of the illness on daily living, the representations of the controllability or curability of the illness, the perception of the illness as a more or less coherent entity, and the emotional impact of illness representations. For the specific need of this study, we focused on two types of illness representations, namely cyclical timeline and curability/controllability.

The IPQ-R proposes scales to measure separately each of these attributes. Timeline is assessed with one scale whereas curability/controllability is evaluated with two scales (i.e., personal control and treatment control). These scales are designed with Likert-type items proposing five response possibilities (score range per item: 1–5). Test-retest reliability for the two illness representations considered in this study (i.e., cyclical timeline, and personal control and treatment control) showed mixed stability at 3 weeks (correlations from 0.46 to 0.72) and 6 months (correlations from 0.35 to 0.57) [42]. A version of the scale in French language is available on its official website (http://www.uib.no/ipq/).

The Brief COPE [43] is a questionnaire assessing 14 coping strategies with two items for each, leading to a total of 28 items. Coping strategies are evaluated using four-point Likert scales. The score range for each coping strategy is 2–8, with high scores indicating frequent use of the strategy in question. In order to investigate the use of instrumental coping strategies before and after DBS (Hypothesis 2), we created a composite variable by adding the scores of three subscales of the Brief COPE, namely active coping (e.g., item 7: “I’ve been taking action to try to make the situation better”), use of instrumental support (e.g., item 10: “I’ve been getting help and advice from other people”), and planning (e.g., item 25: “I’ve been thinking hard about what steps to take”). This composite variable had a score range of 6–24, with higher scores suggesting greater use of instrumental coping strategies. Like the IPQ-R, internal reliability of the Brief COPE scales was mixed with correlations ranging from 0.50 to 0.90 [43].

### 2.4. Statistical Analyses

After computing descriptive statistics for all variables of interest, we attempted to test our two hypotheses by a series of inferential analyses. As these hypotheses were all based on mean comparisons, we initially conducted repeated measures analyzes of variance (rANOVA). Yet, ANOVA deals with missing data through pairwise or listwise deletion, which increases the likelihood to lose information on participants notably in a longitudinal design. For this reason, we ran additional analyses with structural equation modelling (SEM), a statistical method offering better options with regard to missing data. Three nested models were built for model comparison, namely; a level model assuming no change between before and after DBS (i.e., T0 = T1 = T2 = T3); a step model assuming a change between before and after DBS (i.e., T0 ≠ T1 = T2 = T3), and; a model allowing a free slope estimation so that possible changes within post-DBS measurement times can be taken into consideration. This procedure was repeated to address each research hypothesis.

In the present study, the initial sample size (*n* = 45) increases to a potential *n* = 180 because of its longitudinal design comprising four measurement times. Although SEM is generally used with larger sample sizes, it can be applied to smaller samples starting from 30 depending on the model tested [44]. Notably, Bayesian estimation has been found adapted to small samples [45,46]. Thus, we conducted SEM analyses by comparing models estimated; first, with the traditionally used full information maximum likelihood (FIML) and; second, with Bayesian estimation of probability. In order to assess the latter, we examined the following attributes: convergence statistic (acceptable if < 1.002), trace plots stability, convergence in the comparison of first and last third of each parameter’s posterior distribution, stability of autocorrelation plots, and comparison of value estimates with those obtained from FIML analyses. In addition, we provided the deviance information criterion (DIC) and the effective number of parameters (enp) in tables.

All statistical analyses were conducted with IBM SPSS Statistics version 25.0 (IBM Corp., Armonk, NY, USA) and IBM SPSS Amos version 25.0 (IBM SPSS, Chicago, IL, USA). A significance threshold of 0.05 was adopted for inferential statistics. All the data were normally distributed.

## 3. Results

**Hypothesis 1.**  *We tested this hypothesis by using three illness representations variables, that is cyclical timeline, personal control and treatment control*.

First, cyclical timeline representations of PD were stable between pre- and post-DBS assessments as showed by a rANOVA, *F* (2.025, 32.397) = 0.867, *p* = 0.431, *η²* = 0.020, *ω²* = 0.000. Because of sphericity violation (Greenhouse-Geisser *ε* = 0.675), we used Greenhouse-Geisser correction to interpret the analyses.

In contrast with the rANOVA above, SEM analyses showed that patients perceived PD as less cyclical after surgery than before, as demonstrated with significant global mean slope changes in both the step (*b* = −1.482, SE = 0.536, *p* = 0.006) and the free (*b* = −0.556, SE = 0.235, *p* = 0.018) models. The latter model showed a significant change between T0 and T1 (*b* = 3.122, SE = 0.523, *p* < 0.001) with an effect size (*d* = 0.540) estimated as medium to large according to the criteria of Cohen [47]. In FIML estimation, the free model obtained the best statistical fit to the data as depicted in Table 1; yet using Bayesian estimation the step model was better adjusted. Considering that the magnitude of the T1-T2 change was, although significant, very small (*b* = 2.804, SE = 0.528, *p* < 0.001, *d* = 0.053), our data suggest that the change toward less cyclical representations of PD after DBS does not reinforce but stabilizes in the later post-surgical assessment sessions.

Second, patients did not report change in their representation of personal control over PD between pre- and post-DBS assessments as showed by a rANOVA, *F* (3, 48) = 0.044, *p* = 0.987, *η²* = 0.001, *ω²* = 0.000. Because of sphericity violation (Greenhouse-Geisser *ε* = 0.867), we used Huynh-Feldt correction to interpret the analyses.

In the SEM analyses, although the three tested models showed good statistical fit (see Table 2), the step model was significantly better than the level, suggesting that representations of personal control over PD decreased after surgery (*b* = −1.393, SE = 0.627, *p* = 0.026). The free model specified that this diminution had a small to medium effect size in the DBS pre-post transition (*β*_T1_ = 1.776, SE = 0.687, *p* = 0.010, *d* = 0.267) and continued after surgery, albeit with a much smaller amplitude, *β*_T2_ = 2.026, SE = 0.774, *p* = 0.009, *d* = 0.039. Bayesian analyses were adequate for all models, except for the slightly unstable autocorrelation trace plots of the free model.

Third, patients did not report change in their representations of treatment control on PD between pre- and post-DBS assessments as showed by a rANOVA, *F* (2.197, 35.146) = 0.1.195, *p* = 0.318, *η²* = 0.001, *ω²* = 0.000. Because of sphericity violation (Greenhouse–Geisser *ε* = 0.732), we used Greenhouse–Geisser correction to interpret the analyses.

In the SEM analyses, the free model best fitted the data, as shown in Table 2, with significant mean slope change (*b* = −0.312, SE = 0.145, *p* = 0.030) and interindividual variability (*b* = −1.066, SE = 0.411, *p* = 0.010). This suggests that treatment control was perceived as weaker after DBS than before. Yet, slope estimations were not significantly different from 0 at any specific measurement session; in addition, Bayesian analyses showed that the level and step models were better adjusted to the data than the free. Overall, it implies that representations of treatment control did not undergo significant changes over DBS surgery.

**Hypothesis 2.**  *Recourse to instrumental coping was stable over the pre- and post-DBS period, as showed by a rANOVA, F (23, 51) = 2.774, p = 0.051, η² = 0.032, ω² = 0.007. Because of sphericity violation (Greenhouse–Geisser ε = 0.851), we used Huynh–Feldt correction to interpret the analyses*.

In line with this and as summarized in Table 3, the three models designed through SEM did not differ significantly from one another and were very similar in terms of fit indices. However, the Bayesian solution for the free model was not adequate, contrary to these of the level and the step. Detailed analyses of the step model showed that the global mean slope did not change significantly (*b* = −0.562, SE = 0.501, *p* = 0.262).

## 4. Discussion

In this study, we attempted to provide empirical evidence on the question of whether DBS may induce psychological changes using an empirical, inferential approach with patients diagnosed with idiopathic PD. To this end, we targeted two specific variables, namely illness representations and coping strategies.

Based on the existing literature, we had expectations of various kinds of change that DBS would bring in as formulated in two research hypotheses. Yet, globally, our results did not provide confirmation of these expected changes. The first hypothesis, related to illness representations, was partly invalidated as PD was perceived as less cyclical after surgery than before, although patients had lower representations of personal control on PD post-surgically, this latter finding being expected. Regarding the second hypothesis, we observed that the use of instrumental coping strategies was not modified by DBS, which invalidates our initial assumption.

These results suggest that PD remains globally perceived as the same medical entity after DBS, despite the important reduction of motor symptoms induced by brain surgery. Patients’ representations of control on PD decreased after surgery, and this was notably the case for personal control. The notion of control is ethically important as it is associated with autonomy, which, in this context, relates to the patient’s right to decide about his/her treatment [26]. In this regard, feelings of self-estrangement reported from testimonies of operated patients were associated with the impression of losing the control over one’s emotions and capabilities [48]. More generally, a loss of personal control in illness management was expected as it has been well illustrated by testimonies of operated patients [12,49]. In terms of adjustment, this is problematic as subjective perception of control over a chronic disease was associated with adaptive outcomes [33]. This association was notably established in PD patients not treated with DBS [50]. It was however surprising to observe a diminution in representations of treatment control of PD—although this was weak and suggests that, globally, perceptions of treatment control remain similar before and after surgery. One might have indeed imagined that the necessity to rely on neurologists to adapt stimulation parameters would have led to an impression that health professionals may manage the disease efficiently. Possibly related to this latter issue, patients had a less cyclical representation of PD after DBS than before. This suggests that regular appointments with a neurologist to adapt stimulations parameters are not sufficient to create a cyclical representation of PD; however, stabilization of motor fluctuations induced by DBS surgery [51] may have instilled the feelings of a medical condition having become less cyclical after surgery. Representations of PD as becoming more stable after DBS may be related to better psychological well-being, as it was measured in non-DBS patients diagnosed with PD [52].

Finally, we found that use of instrumental coping strategies to deal with stressful situations was not reduced after DBS. This result, which adds to some previous findings [41] but stands in contradiction to others [14,39,40], suggests that DBS does not deeply modify strategies of stress management, the latter being not exclusively dependent on situational issues (e.g., intensity of motor symptoms before vs. after DBS).

Overall, the findings of this study highlight the stability in illness representations and coping strategies throughout the DBS process and rehabilitation. Although significant changes were found here and there, their magnitude was generally small. The only notable exception was representations of PD as being less cyclical after DBS than before. Yet, even in this specific case, magnitude of change should be interpreted with caution. For instance, the implied mean at T0 retrieved from the free model was 13.515 and that at T1 was 11.779. As representations of cyclical timeline are assessed in the IPQ-R with four items, these means correspond to an item mean score of 3.379 at T0 and 2.945 at T1. In other words, representations of cyclical timeline remain around a mean item score of 3 corresponding to a “neither agree nor disagree” response anchor at each measurement time. Thus, even significant and with a medium to large effect size, changes in representations of cyclical timeline remain globally stable over the DBS process and should not be associated with a major alteration in patients’ perception of PD. Interestingly, these small changes were all identified by a critical examination of both FIML and Bayesian estimations in SEM, a method that does not suppress missing data from analyses; on the contrary, rANOVA analyses did not find any significant difference between measurement sessions. Methodologically, this observation implies that SEM remains discriminant with small-size samples, which is a scenario likely to happen frequently in heavy medical situations such as DBS surgery.

Our results should be considered with caution because of limitations inherent to the study design. First, illness representations and coping strategies are not representative of all psychological aspects of the DBS experience. Besides, we decided to focus our analyses on a limited number of illness representations and coping strategies, based on assumptions from the literature and methodological limitations of our experimental design; it remains nevertheless possible that other kinds of illness representations and coping strategies are significantly altered after DBS. Finally, the findings of the current study stand for patients undergoing DBS for PD; it cannot be ruled out that individuals treated with DBS for other medical conditions would experience different outcomes regarding the variables investigated in this research.

## 5. Conclusions

In conclusion, our study suggests that DBS does not induce major psychological changes as measured in terms of illness representations and coping strategies. These conclusions are in line with those of Gilbert et al. [27], who pointed out the lack of current empirical data showing significant changes in personality, self-identity, agency, authenticity, autonomy and self after DBS. Yet, we advocate that future studies continue exploring how psychological variables may impact the acceptation process of life with DBS. In this regard, the burden of normality model may be useful to identify and address the non-motor issues occurring after DBS in PD patients [17]. In addition, and as illustrated in a recent qualitative study [53], considering data related to psychological aspects before surgical implantation may be predictive of post-operative outcome. This implies that a complete psychological assessment taking place before surgery would help clinicians identify risks of developing post-surgical psychosocial complications.

## Figures and Tables

**Table 1:** Comparisons of latent growth curve models estimating growth trajectory for a cyclical representation of PD over an 18-month follow-up (T0–T3).

#	*n*	Mean		SD		Skewness		SE Skewness		Excess Kurtosis		SE Kurtosis		
T0	39	13.564		3.307		−0.473		0.378		0.357		0.741		
T1	30	11.833		3.495		0.144		0.427		−0.693		0.833		
T2	20	11.600		3.050		0.065		0.512		−0.616		0.992		
T3	23	12.522		3.475		−0.156		0.481		−0.277		0.935		
#	Tested models	*χ^2^*	df	*p*-value	*χ^2^*/df	CFI	TLI	RMSEA (90% CI), pclose	Model comparison	Δ *χ^2^*	Δ df	*p*-value	*DIC*	*enp*
1	Level	35.053	11	<0.001	3.187	0.281	0.346	0.226 (0.144–0.311), 0.001	1 vs 2	7.385	1	0.007	416.67	2.78
2	Step	27.668	10	0.002	2.767	0.472	0.472	0.203 (0.115–0.295), 0.005	1 vs 3	20.173	5	0.001	411.16	3.78
3	Free	14.880	6	0.021	2.480	0.734	0.557	0.186 (0.067–0.307), 0.036	2 vs 3	12.788	4	0.012	406.08	7.05

Note: SD = standard deviation, SE = standard error, *p* = probability, *χ2* = chi-squared, df = degrees of freedom, CFI = comparative fit index, pclose = probability that the RMSEA value is below .05, TLI = Tucker-Lewis index, RMSEA = root mean square error of approximation, CI = confidence intervals, DIC = deviance information criterion, enp = effective number of parameters

**Table 2:** (**a**)Comparisons of latent growth curve models estimating growth trajectory for the representation of a personal control of PD over an 18-month follow-up (T0–T3). (**b**) Comparisons of latent growth curve models estimating growth trajectory for the representation of a control of PD by treatment over an 18-month follow-up (T0–T3).

(a)														
#	*n*	Mean		SD		Skewness	SE Skewness		Excess Kurtosis		SE Kurtosis			
T0	39	20.026		4.374		−0.945	0.378		1.274		0.741			
T1	30	18.900		4.302		−0.531	0.427		0.745		0.833			
T2	20	19.300		4.256		−0.604	0.512		−0.642		0.992			
T3	23	17.609		4.356		−0.209	0.481		−0.973		0.935			
#	Tested models	*χ^2^*	df	*p*-value	*χ^2^*/df	CFI	TLI	RMSEA (90% CI), pclose	Model comparison	Δ *χ^2^*	Δ df	*p*-value	*DIC*	*enp*
1	Level	9.675	11	0.560	0.880	1.000	1.055	0.000 (0.000–0.145), 0.648	1 vs 2	4.799	1	0.028	460.35	2.82
2	Step	4.876	10	0.89	0.488	1.000	1.232	0.000 (0.000–0.072), 0.928	1 vs 3	8.586	5	0.127	457.35	3,76
3	Free	1.089	6	0.982	0.181	1.000	1.371	0.000 (0.000–0.000), 0.986	2 vs 3	3.787	4	0.436	461.72	7.20
**(b)**														
#	N	Mean		SD		Skewness	SE skewness		Excess kurtosis		SE kurtosis			
T0	39	16.923		3.800		0.342	0.378		−0.129		0.741			
T1	30	15.333		4.063		−0.986	0.427		0.826		0.833			
T2	20	16.650		3.588		−0.526	0.512		−0.629		0.992			
T3	23	15.739		2.942		−1.139	0.481		2.512		0.935			
#	Tested models	*χ^2^*	df	*p*-value	*χ^2^*/df	CFI	TLI	RMSEA (90% CI), pclose	Model comparison	Δ *χ^2^*	Δ df	*p*-value	*DIC*	*enp*
1	Level	17.399	11	0.097	1.582	0.649	0.681	0.116 (0.000–0.215), 0.152	1 vs 2	3.094	1	0.079	432.83	2.78
2	Step	14.305	10	0.160	1.431	0.764	0.764	0.100 (0.000–0.207), 0.228	1 vs 3	11.61	5	0.041	431.77	3.79
3	Free	5.789	6	0.447	2.480	0.734	0.557	0.000 (0.000–0.194), 0.516	2 vs 3	8.516	4	0.074	-	-

**Table 3:** Comparisons of latent growth curve models estimating growth trajectory for a cyclical representation of PD over an 18-month follow-up (T0-T3).

#	*n*	Mean		SD		Skewness		SE Skewness		Excess Kurtosis		SE Kurtosis		
T0	42	14.000		4.283		0.274		0.365		−0.408		0.717		
T1	31	13.097		3.581		0.051		0.421		−0.659		0.821		
T2	24	14.333		3.795		−0.750		0.472		0.063		0.918		
T3	24	13.417		3.944		−0.047		0.472		−0.875		0.918		
#	Tested models	*χ^2^*	df	*p*-value	*χ^2^*/df	CFI	TLI	RMSEA (90% CI), pclose	Model comparison	Δ *χ^2^*	Δ df	*p*-value	*DIC*	*enp*
1	Level	17.093	11	0.105	1.554	0.868	0.955	0.112 (0.000–0.210), 0.164	1 vs 2	1.245	1	0.265	457.12	2.85
2	Step	15.848	10	0.104	1.585	0.874	0.874	0.115 (0.000–0.217), 0.160	1 vs 3	7.326	5	0.198	457.77	3.80
3	Free	9.767	6	0.135	1.628	0.919	0.551	0.119 (0.000–0.250), 0.185	2 vs 3	6.081	4	0.193	-	-
